# Exploring Moderators of the Effect of High vs. Low-to-Moderate Intensity Exercise on Cardiorespiratory Fitness During Breast Cancer Treatment – Analyses of a Subsample From the Phys-Can RCT

**DOI:** 10.3389/fspor.2022.902124

**Published:** 2022-07-12

**Authors:** Ann Christin Helgesen Bjørke, Laurien M. Buffart, Truls Raastad, Ingrid Demmelmaier, Andreas Stenling, Karin Nordin, Sveinung Berntsen

**Affiliations:** ^1^Department of Sport Science and Physical Education, University of Agder, Kristiansand, Norway; ^2^Department of Public Health and Caring Sciences, Uppsala University, Uppsala, Sweden; ^3^Department of Physiology, Radboud University Medical Center, Radboud Institute for Health Sciences, Nijmegen, Netherlands; ^4^Exercise Medicine Research Institute, Edith Cowan University, Joondalup, WA, Australia; ^5^Department of Physical Performance, Norwegian School of Sport Sciences, Oslo, Norway; ^6^Department of Psychology, Umeå University, Umeå, Sweden

**Keywords:** cardiorespiratory fitness (CRF), breast cancer, moderators, intensity, age, exercise adherence, endurance and strength training

## Abstract

**Introduction:**

The results from the physical training and cancer randomized controlled trial (Phys-Can RCT) indicate that high intensity (HI) strength and endurance training during (neo-)adjuvant cancer treatment is more beneficial for cardiorespiratory fitness (CRF, measured as peak oxygen uptake [VO_2_peak]) than low-to-moderate intensity (LMI) exercise. Adherence to the exercise intervention and demographic or clinical characteristics of patients with breast cancer undergoing adjuvant treatment may moderate the exercise intervention effect on VO_2_peak. In this study, the objective was to investigate whether baseline values of VO_2_peak, body mass index (BMI), time spent in moderate- to vigorous-intensity physical activity (MVPA), physical fatigue, age, chemotherapy treatment, and the adherence to the endurance training moderated the effect of HI vs. LMI exercise on VO_2_peak.

**Materials and Methods:**

We used data collected from a subsample from the Phys-Can RCT; women who were diagnosed with breast cancer and had a valid baseline and post-intervention VO_2_peak test were included (*n* = 255). The exercise interventions from the RCT included strength and endurance training at either LMI, which was continuous endurance training at 40–50% of heart rate reserve (HRR), or at HI, which was interval training at 80–90% of HRR, with similar exercise volume in the two groups. Linear regression analyses were used to investigate moderating effects using a significance level of *p* < 0.10. Statistically significant interactions were examined further using the Johnson–Neyman (J-N) technique and regions of significance (for continuous variables) or box plots with adjusted means of post-intervention VO_2_peak (for binary variables).

**Results:**

Age, as a continuous variable, and adherence, dichotomized into < or > 58% based on median, moderated the effect of HI vs. LMI on CRF (*B* = −0.08, 95% CI [−0.16, 0.01], *p*_*interaction*_ = 0.06, and *B* = 1.63, 95% CI [−0.12, 3.38], *p*_*interaction*_ = 0.07, respectively). The J-N technique and regions of significance indicated that the intervention effect (HI vs. LMI) was positive and statistically significant in participants aged 61 years or older. Baseline measurement of CRF, MVPA, BMI, physical fatigue, and chemotherapy treatment did not significantly moderate the intervention effect on CRF.

**Conclusion:**

Women with breast cancer who are older and who have higher adherence to the exercise regimen may have larger effects of HI exercise during (neo-)adjuvant cancer treatment on CRF.

## Introduction

Although improved diagnostic techniques and treatments have increased the survival rates in the recent decades (Torre et al., 2016; Bray et al., 2018), individuals with a history of cancer struggle with reduced physical function and quality of life due to various side effects (Banzer et al., 2014). Women treated for breast cancer often experience impaired cardiorespiratory fitness (CRF) (Jones et al., 2012; Peel et al., 2014), which is associated with increased cardiovascular (Jones et al., 2007, 2012) and cancer-specific mortality (Schmid and Leitzmann, 2015). In both healthy individuals and in patients with cardiovascular disease, CRF, measured as peak oxygen uptake (VO_2_peak), is an important clinical predictor of overall mortality (O'Neill et al., 2005; Kodama et al., 2009; Ekblom-Bak et al., 2019).

The results from the physical training and cancer randomized controlled trial (Phys-Can RCT) indicated that high intensity (HI) strength and endurance training during cancer treatment are associated with more beneficial changes in VO_2_peak, compared to low-to-moderate intensity (LMI) exercise (Demmelmaier et al., 2021). When comparing two different exercise intensities, adherence to the exercise intervention(s) is an important factor for understanding the cause-and-effect relationship (Hecksteden et al., 2018). In the Phys-Can RCT, adherence to the endurance training was higher in the LMI group than in the HI exercise group (Demmelmaier et al., 2021). Consequently, the effect of HI vs. LMI exercise on CRF may be moderated by adherence to the intervention, but also by demographic or clinical characteristics of the patients (Courneya et al., 2004; Jackson et al., 2009; Shang et al., 2012; Lakoski et al., 2013; Buffart et al., 2018; Sweegers et al., 2019; Zeiher et al., 2019; Wiestad et al., 2020).

In a meta-analysis of individual patient data, larger effects of exercise interventions on CRF were found in younger patients (Sweegers et al., 2019), and in patients with higher CRF prior to the exercise intervention (Buffart et al., 2018). However, this meta-analysis did not examine whether exercise response to different intensities varied in various subgroups (Buffart et al., 2018; Sweegers et al., 2019). Therefore, it is not clear whether the observed intervention effect (HI vs. LMI) on CRF varies by clinical or demographic characteristics in the meta-analysis. In a meta-analysis including healthy, young to middle-aged adults, HI interval training produced larger improvements in CRF compared to LMI continuous endurance training, with greater benefits for the less fit adults, those with a lower baseline CRF (Milanovic et al., 2015). Objective measures of baseline physical activity level and VO_2_peak before an exercise intervention may (partly) represent a person's physical activity and exercise history and, therefore, are relevant to consider as the moderators of the intervention effect on CRF (Zeiher et al., 2019). In addition to low CRF at baseline, people with a history of low physical activity or exercise levels may respond differently to HI or LMI exercise. As people get older, physical activity levels and CRF decrease (Jackson et al., 2009; Westerterp, 2018), and in the general adult population, age is found to be a determinant for CRF (Zeiher et al., 2019). Furthermore, body mass index (BMI) has also been found to be associated with CRF in the healthy population (Zeiher et al., 2019).

Additionally, chemotherapy may also directly and indirectly influence the exercise-induced change in CRF (Lakoski et al., 2013; Wiestad et al., 2020). Physical fatigue may affect how well people manage to perform the prescribed exercise and consequently indirectly affect the exercise-induced change in CRF (Christensen et al., 2018).

The objective of this study was to investigate whether baseline values of VO_2_peak, BMI, time spent in moderate- to vigorous-intensity physical activity (MVPA), physical fatigue, age, chemotherapy treatment, and the adherence to the endurance training moderated the effect of 6 months of HI vs. LMI exercise during breast cancer treatment on VO_2_peak.

## Materials and Methods

### Design and Participants

The study protocol (Berntsen et al., 2017) and the primary study results from the Phys-Can RCT, a multicenter 2 × 2 factorial design trial, have been published previously (Demmelmaier et al., 2021), and this study is based on secondary analyses of data from this study. Phys-Can RCT was approved by the Regional Ethical Review Board in Uppsala (Dnr 2014/249) and registered in ClinicalTrials.gov (TRN = NCT02473003, Oct 2014) (Berntsen et al., 2017; Demmelmaier et al., 2021). The recruitment process has been described previously in detail (Demmelmaier et al., 2021). Briefly, eligible patients who were newly diagnosed with breast, colorectal, or prostate cancer and scheduled to undergo (neo-)adjuvant cancer treatment were recruited at university hospitals in three cities of Sweden (Lund/Malmö, Linköping, and Uppsala) between March 2015 and April 2018. Participants were stratified by study site and randomized to one of four intervention groups: HI vs. LMI exercise *with* or *without* additional behavioral change support. Exclusion criteria were stage IIIb–IV breast cancer, inability to perform basic activities of daily living, cognitive disorders, severe psychiatric disease, or other disabling conditions that might contraindicate HI exercise, treatment for an additional ongoing malignant disease, and BMI < 18.5 kg/m^2^ or pregnancy.

For this study, only patients with breast cancer and a VO_2_peak test that met the criteria for accepted tests at baseline and post-intervention were included, resulting in a total number of 255 participants. All measurements and questionnaires were completed before and after the exercise intervention.

### Exercise Interventions

The exercise program has been described in detail in previous publications (Berntsen et al., 2017; Demmelmaier et al., 2021). Briefly, all participants performed supervised strength training and home-based endurance training for 6 months while undergoing (neo-)adjuvant cancer treatment. The resistance training prescription in all four groups consisted of six machine-based exercises performed two times a week, with different intensity, sets, and repetitions based on intensity group. In the HI exercise groups, participants performed their repetitions alternating between 3 sets × 6 repetitions maximum (RM) and 3 sets × 10 RM until failure. In the LMI groups, participants performed their repetitions alternating between 3 sets × 12 repetitions and 3 sets × 20 repetitions until 50% of maximum. The endurance training for the HI exercise groups consisted of two times weekly interval training at 80–90% of heart rate reserve (HRR) during the 2-min-long intervals with 1 min of “rest” in between intervals, with the number of intervals progressed from 5 to 10 over the course of the program. In the LMI groups, 150 min of continuous exercise at 40–50% of HRR was prescribed weekly. The endurance training mode was chosen by participants according to their preference and could be running, cycling, walking uphill, or any other endurance-based activity. After a familiarization period introducing the heart rate monitor and how to use the Borg Scale (Borg, 1970) to monitor exercise intensity, the home-based endurance training under weekly guidance by a coach began. Half of the participants received additional support to facilitate adherence to the respective exercise programs and to maintain physical activity levels after the completion of the interventions. This behavioral change support included in the Phys-Can RCT has been described previously in detail (Demmelmaier et al., 2021).

### Measurement of Cardiorespiratory Fitness

The main outcome in this study, CRF, was measured in exercise laboratories as VO_2_peak with gas exchange. A full description of methods, criteria, and the equipment used for measuring VO_2_peak in the Phys-Can RCT, can be found elsewhere (Bjørke et al., 2020). Briefly, participants walked or ran to exhaustion on a treadmill using a modified Balke protocol (Edvardsen et al., 2013). For this analysis, only the tests meeting two of the three following criteria for an accepted exercise test were included: (a) the tester judged the test as maximal using their subjective evaluation of body language, facial expression, hyperventilation, and other signs reflecting that a maximal effort had been given; (b) a Borg rating of perceived exertion, (Borg et al., 1987) rating ≥ 17; and (c) respiratory exchange ratio (RER) ≥ 1.1 (Bjørke et al., 2020).

### Assessment of Potential Moderators

Demographic and clinical variables were collected by self-reported validated questionnaires and through medical records. All of the assessment methods have been described in previous publications (Berntsen et al., 2017; Bjørke et al., 2020; Demmelmaier et al., 2021). Briefly, health-related quality of life was assessed with the European Organization for Research and Treatment of Cancer (EORTC) QLQ-C30, range 0 (low quality of life) to 100 (high quality of life) (Aaronson et al., 1993). Physical fatigue was assessed with the Multidimensional Fatigue Inventory (MFI) (Smets et al., 1995) with a subscale ranging from 4 (low fatigue) to 20 (high fatigue).

Physical activity level at baseline was assessed with a SenseWear Armband Mini (SWA), a tri-axial monitor previously validated in cancer survivors (Cereda et al., 2007). The Professional 8.1 Software was used to calculate SWA wear time and minutes spent in MVPA, using the established cutoff value of at least 3.0 metabolic equivalent task values (Garber et al., 2011). Weekly time spent in MVPA levels was calculated by summing minutes for each valid day on which the criterion for MVPA was met.

The calculation methods for exercise adherence have been described in detail in previous publications (Demmelmaier et al., 2021) and in accordance with previous research (Nilsen et al., 2018). Briefly, exercise adherence was calculated as performed exercise divided by prescribed exercise. The calculation represented exercise volume (frequency, intensity, and time) and resulted in one value (0–100%) for resistance training and one value (0–100%) for endurance training for each participant. In this study, the variable representing adherence to the endurance training is included. When adherence to the endurance training was included by statistical or/and illustrative purposes, the median was used to dichotomize into two groups, because of the non-normal distribution of the variable.

### Statistical Analyses

Except for illustrations of figures, all statistical analyses were performed using the Statistical Package for the Social Sciences version 25.0 (SPSS, IBM Corp. Released 2017. IBM SPSS Statistics for Windows, version 25.0. Armonk, New York, USA).

Baseline descriptive including demographic, clinical, or behavioral characteristics is presented as mean and standard deviation (*SD*) for continuous variables and proportions as number (*n*) and percentage (%) for categorical variables.

**Figure 1:**
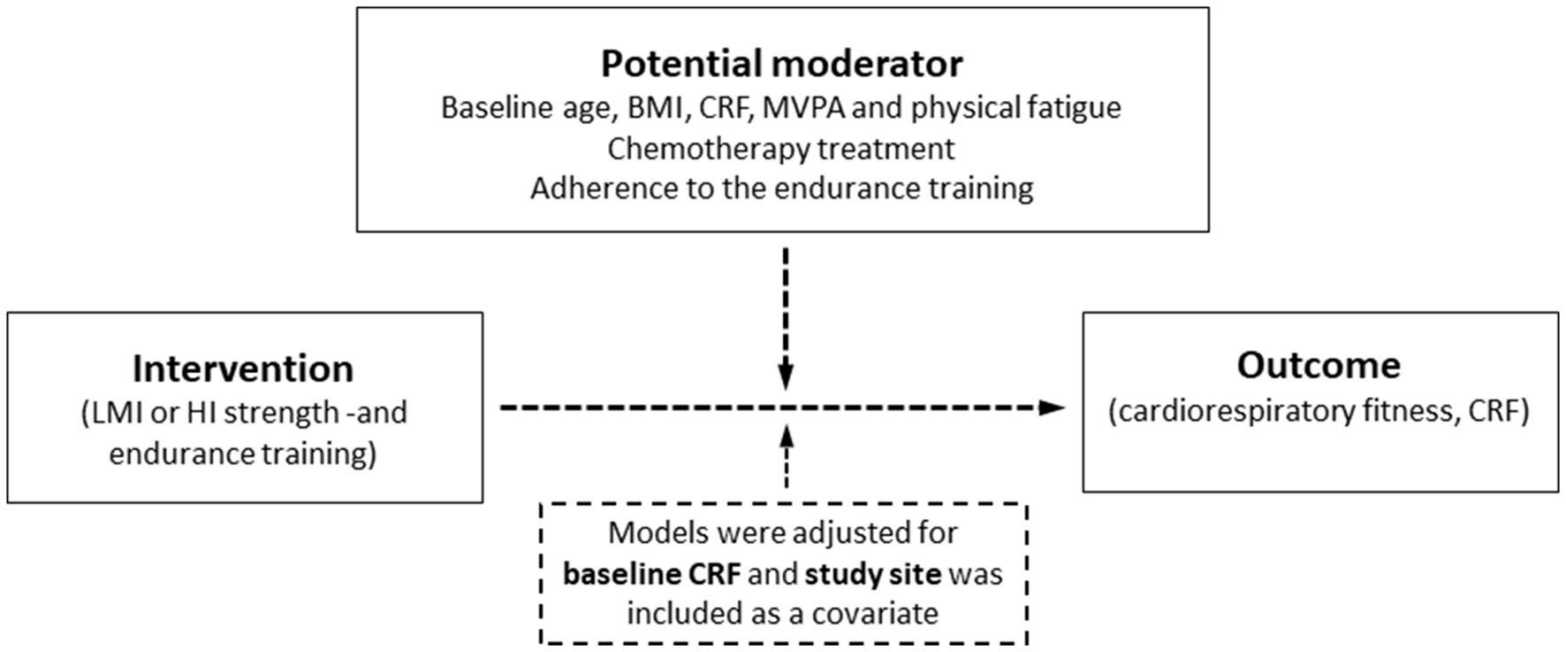
The hypothesized moderation model. Intervention effect (mean difference in change between low-to-moderate intensity (LMI) and high intensity (HI) training) on peakimal oxygen uptake (VO_2_peak) post-intervention. The potential moderator variables investigated were baseline age, body mass index (BMI), cardiorespiratory fitness (CRF), hours in moderate-to-vigorous physical activity (MVPA), physical fatigue, chemotherapy treatment (yes/no), and adherence to the endurance training. Each of the potential moderator variables was included in the regression model one at a time, resulting in a total of seven models.

Continuous moderators were kept as continuous if the variables were normally distributed (by visual inspection of plots) and dichotomized if not. Linear regression analyses were used to investigate potential moderators of the effect of HI vs. LMI exercise on CRF (refer to Figure 1). In the model, post-intervention VO_2_peak was regressed on the intervention (HI vs. LMI exercise) while adjusting for baseline VO_2_peak. Study site was included as a covariate in the models, due to the possibility that patients from the three sites may have been receiving some variations regarding treatment regimes, test-personnel, and coaches. Moderating effects were examined by adding the moderator variable and its interaction term with the intervention variable into the regression model. Because the interaction effects were exploratory and intended to generate hypotheses for future research, we used an alpha level of 0.10 for the interaction terms (Durand, 2013). The results are presented as unstandardized regression coefficients (*b*) and 95% CI.

Models with continuous moderators were estimated using the PROCESS macro-version 4.0 for SPSS (Hayes, 2022). PROCESS uses ordinary least squares regression analysis when predicting continuous variables and can be used to probe and visualize interactions using the Johnson–Neyman (J-N) technique (Lin, 2020). The J-N technique allows the assessment of whether and how a continuous variable moderates the relationship between exercise intensity and VO_2_peak after the intervention by indicating a “region of significance,” which is the case if the upper and lower CIs are completely below or above zero. A J-N plot was made by using the coefficients of the regression model in Microsoft^®^ Excel^®^ for Microsoft 365, version 2202 to visualize the moderated effect of the intervention (HI vs. LMI exercise) on VO_2_peak.

For visualizations of statistically significant binary moderators such as chemotherapy treatment, the estimated margin of means of VO_2_peak post-intervention with adjustment for baseline VO_2_peak and study site was reported within subgroups of the moderator. Dichotomization of continuous variables was carried out using median-split or by cut points derived through J-N plots, which were made as the preferable choice. These figures related to the binary variables were made using GraphPad Prism, version 7.00 for Windows (GraphPad Software, La Jolla California, USA, www.graphpad.com).

## Results

Baseline characteristics of the participant with breast cancer with a valid pre- and post-intervention VO_2_peak test, reported for the LMI and the HI group, are presented in Table 1.

**Table 1:** Baseline characteristics of participants with breast cancer in Phys-Can RCT. Data are presented as mean (±SD) or *n* (%).

**Characteristic**	**LMI (*n =* 127)**	**HI (*n =* 128)**
*Study site, n (%)*		
Linköping	30 (24)	24 (19)
Lund	56 (44)	54 (42)
Uppsala	41 (32)	50 (39)
Age, years, mean (SD)	53 (10)	55 (11)
Living with a partner, *n* (%)	99 (78)	95 (74)
Completed university, *n* (%)	90 (71)	83 (65)
*Sick-leave, n (%)*		
100% sick-leave	49 (39)	46 (36)
75, 50, or 25% sick-leave	7 (6)	9 (7)
*Weight status, n (%)*		
BMI – normal (18.5–24.9)	65 (51)	77 (60)
BMI – overweight (25–29.9)	49 (39)	32 (25)
BMI – obesity (≥ 30)	13 (10)	19 (15)
Current smoker, *n* (%)	6 (2)	0 (0)
*Treatment, n (%)*		
Chemotherapy	89 (70)	84 (66)
No chemotherapy	38 (30)	44 (34)
*EORTC QLQ C30, mean (SD)*		
Global health status/QoL	61 (19)	64 (20)
Physical function	87 (14)	89 (13)
Physical fatigue, MFI, mean (SD)	12 (4)	12 (4)
Kinesiophobia, mean (SD)	23 (5)	23 (5)
MVPA, hours/day, mean (SD)	1.3 (1.0)	1.3 (0.9)
VO_2_peak (ml/kg/min), mean (SD)	31.3 (7.1)	31.5 (7.3)

*BMI, body mass index; EORTC QLQ C30, European Organization for Research and Treatment of Cancer, quality of life of cancer patients; QoL, quality of life; MFI, Multidimensional Fatigue Inventory; Tampa, The Tampa Scale of Kinesiophobia; MVPA, moderate- to vigorous-intensity physical activity; VO_2_peak, peak oxygen uptake (ml per kilogram per min)*.

### Potential Moderating Variables

**Figure 2:**
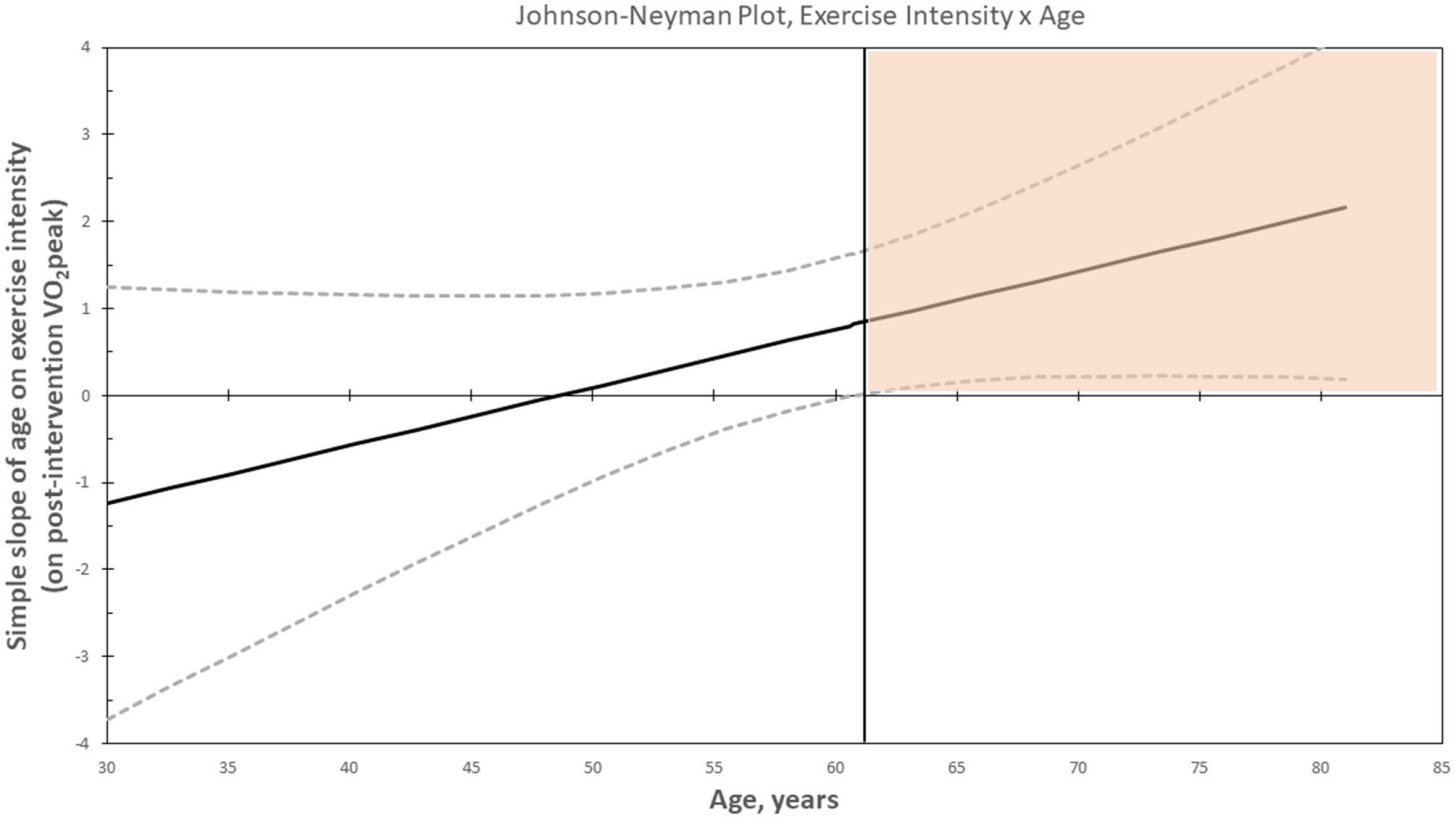
Johnson–Neyman plot showing the simple slope (with the 95% confidence intervals) of age on exercise intensity on post-intervention VO_2_peak. Colored area represents the region of significance (*p* < 0.05).

**Figure 3:**
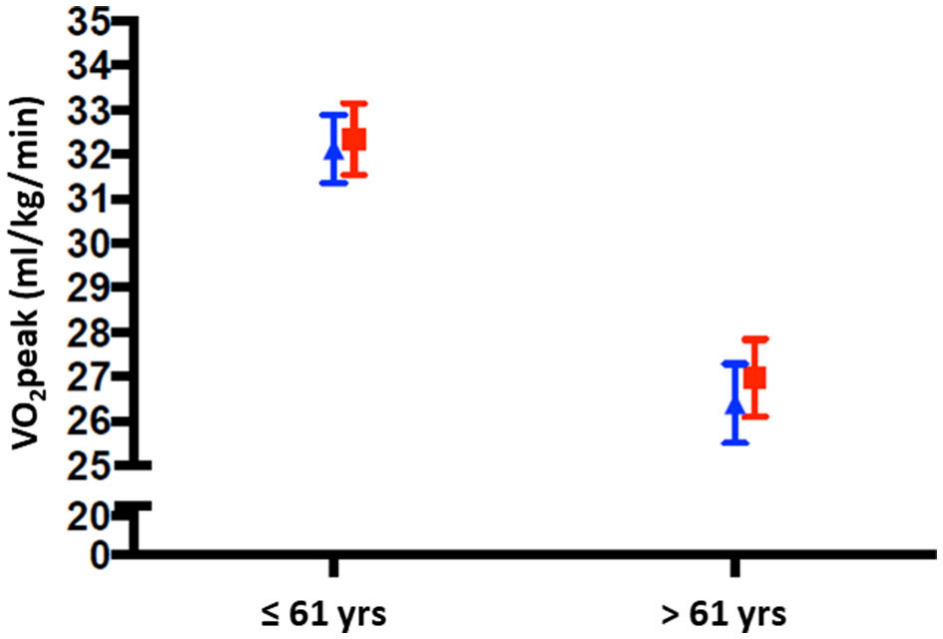
VO_2_peak post-intervention below and over the age cutoff (61 years), presented within the two intensity groups LMI (blue triangle) and HI (red squares). Means are adjusted for VO_2_peak at baseline and study site is included as covariate.

The regression coefficients of each of the interaction terms with 95% confidence intervals (CIs) are presented in Table 2 (a complete table is presented in the Supplementary Table S1). Age moderated the effect of HI vs. LMI exercise on CRF (*B* = −0.08, 95% CI [−0.16, 0.01], *p*_*interaction*_ = 0.06). The J–N plot illustrated that the intervention effect (HI vs. LMI exercise) was positive, in favor of HI, and statistically significant in patients aged 61 years or older, *n* = 68, as visualized in the colored area in the plot (Figure 2), whereas the intervention effect was not statistically significant in patients younger than 61 years, *n* = 187. These results are further visualized in Figure 3, where the adjusted post-intervention VO_2_peak within LMI and HI exercise groups and within above and below 61 years is presented. The mean exercise adherence in women aged ≤ 61 years was 65% (SD 32) and 52% (SD 32) for the LMI and HI exercise groups, respectively. For patients aged ≥62, the mean exercise adherence was 66% (SD 31) and 42% (SD 30) for the LMI and HI exercise groups, respectively (Supplementary Figure S1). Chemotherapy treatment was administered in 75% of the younger women and in 49% of the older women.

**Table 2:** Results of the analyses examining potential moderators of HI vs. LMI exercise on VO_2_peak.

**Model**	**Potential moderator variable**	**b_**interaction**_**	**95% CI**	***p*** **-value**
1	Age, years	−0.08	−0.16, 0.01	**0.06**
2	BMI, weight in kg/height in m^2^	−0.04	−0.24, 0.15	0.65
3	Physical fatigue, MFI	−0.01	−0.23, 0.21	0.94
4	Chemotherapy treatment (yes/no)	0.03	−1.77, 1.83	0.97
5	MVPA, hours/wk	−0.61	−1.57, 0.34	0.21
6	Baseline CRF (VO_2_peak, ml/kg/min)	0.09	−0.03, 0.21	0.15
7	Exercise adherence (< /> median adherence)	1.63	−0.12, 3.38	**0.07**

*BMI, body mass index; MFI, Multidimensional Fatigue Inventory; MVPA, moderate to vigorous physical activity; treatment, cytostatic vs. no cytostatic; CRF, cardiorespiratory fitness. Median adherence = 58%. Significance level was set to p < 0.10 for further exploration*.

**Figure 4:**
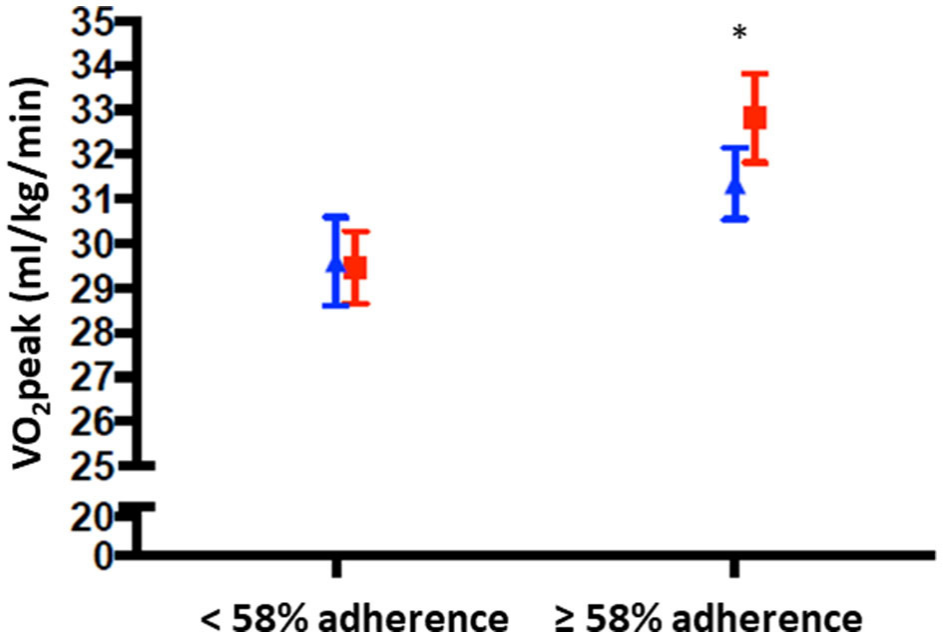
VO_2_peak post-intervention below and over median adherence to the endurance training (58%), presented within the two intensity groups LMI (blue triangle) and HI (red squares). Means are adjusted for VO_2_peak at baseline and study site is included as covariate. * = *p* < 0.01.

Adherence to the endurance training moderated the intervention effect of HI vs. LMI exercise on CRF (*B* = 1.63, 95% CI [−0.12, 3.38], *p*_*interaction*_ = 0.07), with larger effects in patients who adhered above 58% (Figure 4).

Baseline CRF, MVPA, BMI, physical fatigue, and chemotherapy treatment did not significantly moderate the effect of HI vs. LMI exercise on CRF (Table 2 and Supplementary Table S1).

## Discussion

Both age and adherence to the endurance training were found to moderate the intervention effect of HI vs. LMI exercise on CRF with significant beneficial effects in patients aged 61 years or older and in patients with adherence >58%.

Demographic and clinical variables did not moderate the intervention effect of HI vs. LMI exercise on CRF.

Our finding that age moderated the intervention effect on CRF in a cancer population is in line with the previous studies (Courneya et al., 2008; Sweegers et al., 2019). However, these studies did not investigate the modifying role of age in combination with exercise intensity (Courneya et al., 2008; Sweegers et al., 2019), making it less relevant for comparison with the findings in this study. Nevertheless, the general trend in both of these studies was that young patients responded better to the intervention than older patients irrespective of exercise intensity. In a meta-analysis, Sweegers et al. (2019) reported the intervention effect to be significantly larger when compared to usual care for patients younger than 50 years (Sweegers et al., 2019). Additionally, in the RCT published by Courneya et al. (2008) patients younger than 50 years were found to have a larger improvement in their VO_2_peak after performing aerobic exercise training compared to usual care. This was noted in comparison with women above 50 years, in which there was no difference between exercise and usual care (Courneya et al., 2008). We found that this moderator effect of age is not only the case when comparing exercise vs. usual care but also for HI vs. LMI exercise. This means that older women may achieve a more beneficial change in their CRF if they exercise at HI, compared to LMI.

The difference in intervention effects by age could not be explained by the differences in adherence to the endurance training, as women aged above 61 years had markedly lower average adherence in the HI exercise group (43%) compared to the LMI exercise group (66%). The same trend was observed for women under the age of 61 years, but adherence to HI was higher in the younger group: 52% in the HI exercise group and 65% in the LMI exercise group (Supplementary Figure S1).

Older women have lower CRF compared to younger women (Jackson et al., 2009). When adding the literature related to the fact that less fit adults may achieve improvements in CRF at a relatively lower exercise intensity level than more fit adults (Milanovic et al., 2015), our findings are somewhat contradictory to the existing literature. Our results indicate that it is the less fit women (the oldest) who gain CRF benefits from exercising at higher intensities than lower intensities. For women younger than 61 years, the exercise intensity did not appear to affect the CRF results to the same degree. Importantly, CRF is relative to age and should be reported as a percentage of the age predicted VO_2_peak. There were no differences between the young and old groups, with the young group (mean age 49 years) averaging 84% of predicted VO_2_peak, and the old group (mean age 68 years) averaging 85% of predicted VO_2_peak, when using data from the Norwegian HUNT study as a reference for healthy women in different age groups (Loe et al., 2013).

We did not find chemotherapy treatment as a binary variable (yes/no) to be a moderator of the intervention effect in this study. Although others have found more intense treatment such as chemotherapy in addition to radiotherapy, to moderate the intervention effect when compared to a control group (Kalter et al., 2015), our study may have been underpowered to detect a moderation effect between two exercise intensities. The fact that women aged above 61 years in Phys-Can were more seldom prescribed breast cancer treatment including chemotherapy (49 vs. 75%) is relevant to mention in relation to our finding of age being a moderator for the intervention effect on CRF. That older patients less often receive chemotherapy agrees with other studies as well (August et al., 1994; Partridge, 2021).

Interestingly, although chemotherapy treatment itself did not moderate the intervention effect, the fact that the older women received less chemotherapy may be a part of the explanation for why age was found to moderate the intervention effect on VO_2_ peak in favor of HI exercise. Although cancer itself in combination with surgery, radiotherapy, and endocrine treatment can impair CRF, the chemotherapy-induced toxicity caused by the generation of reactive oxygen species and the induction of cardiac myocyte apoptosis or necrosis is known to be the most harmful treatment, especially if the therapy is anthracycline-based (Chen et al., 2007; Shi et al., 2011; Suter and Ewer, 2013). There may be other mechanisms for why chemotherapy impairs CRF, for example, anemia, dehydration, and impairment of pathways within muscle cells which again impair the O_2_ extraction from the blood to the muscles (Jones et al., 2009; Christensen et al., 2018). Consequently, it may be more physiologically difficult for younger women to gain the beneficial effect of HI vs. LMI exercise because more of them receive chemotherapy.

Another aspect to acknowledge relating to the finding of age as a moderator is the wide CIs, especially for the youngest and oldest participants, due to the few participants (in the oldest and youngest age) and the large variations in VO_2_peak. Wide CIs indicate that the estimates are less precise, and when adding the observation (in the J-N plot) that the lower limit of the CI is just above zero, the finding of age as a moderator should be interpreted with caution. In summary, there is a strong indication that age may moderate the effect of the intervention on VO_2_peak after being randomized to HI or LMI exercise for 6 months during breast cancer treatment. However, we suspect this finding to be somewhat disturbed by variations in chemotherapy treatment across the age span.

### Adherence to the Endurance Training

Adherence to the endurance training was found to moderate the intervention effect on VO_2_peak, where women who adhered above the median level had a significantly higher VO_2_peak if they had been performing HI exercise, compared to LMI exercise.

In the Phys-Can RCT (Demmelmaier et al., 2021), adherence to the endurance training was higher in the LMI exercise group compared to the HI group and also confirmed when including only the women with breast cancer, as in this study (Supplementary Figure S1). Higher adherence to an intervention including lower exercise intensity has also been reported in men with prostate cancer (Nilsen et al., 2018). We found that younger women have a smaller difference in adherence between intensity groups compared to the oldest women. This observation, in combination with older age having a stronger intervention effect, underlines the importance of including strategies to optimize adherence in the oldest participants.

In total, the finding related to exercise adherence substantiates the notion that participants need to perform the training with a certain diversity in intensity to produce a difference in effect on VO_2_peak. HI exercise is most beneficial for improving VO_2_peak in patients undergoing treatment for cancer, given that the patients can manage to complete the HI training.

The fact that demographic and clinical variables did not moderate the intervention effect of HI vs. LMI exercise on CRF implies that if women have variations within baseline values of BMI, MVPA, CRF, or physical fatigue and are scheduled for chemotherapy treatment or not, we cannot specify one exercise intensity over the other. Consequently, women may choose either LMI or HI exercise according to their own preferences.

### Strengths and Limitations

Regarding the exercise intervention in the Phys-Can RCT, there was thorough monitoring of exercise frequency, intensity, type, and duration to ensure that the exercise volume was similar in the two intensity groups. Still, it should be mentioned that the reporting method did differ between HI and LMI exercise groups. In the LMI group, we could not calculate adherence to frequency, intensity, time, and type of endurance training, refer to Mazzoni et al. (2020) for a discussion related to adherence reporting (Mazzoni et al., 2020). Another strength of the Phys-Can RCT was that the participants walked or ran on a treadmill when performing the VO_2_peak test, which is the gold standard and a direct method to measure CRF. We also used criteria for including or excluding valid and accepted tests.

To our knowledge, we have performed the first investigation of potential moderators for this specific intervention effect (HI vs. LMI strength and endurance training) on CRF in women undergoing (neo-)adjuvant breast cancer treatment. By investigating these potential moderators, we were able to identify possible subgroups of patients with breast cancer who could benefit most from exercising at HI vs. LMI. Our findings that many of the investigated variables do not moderate the intervention effect, according to being randomized to HI or LMI exercise, suggest that most women may choose their preferable exercise intensity. The difference in CRF is not large between the LMI and HI exercise groups, although the HI group seems to benefit more than the LMI group (Demmelmaier et al., 2021). However, among those with higher adherence, a larger intervention effect was observed for the HI exercise group compared to the LMI group.

## Conclusion

The primary finding in this study was that age moderated the intervention effect, and more specifically, women who are diagnosed with breast cancer when they are older may reap greater benefits to their CRF from HI exercise than LMI exercise during (neo-)adjuvant cancer treatment. Adherence to the endurance training is important for achieving a beneficial change in CRF of exercising at HI, compared to LMI. Therefore, strategies to improve exercise adherence in the population of women undergoing (neo-)adjuvant breast cancer treatment are important.

Baseline levels of BMI, MVPA, CRF, and physical fatigue, in addition to chemotherapy treatment, do not seem to moderate the intervention effect on CRF by exercising for 6 months at LMI levels vs. HI levels during (neo-)adjuvant breast cancer treatment. Therefore, if women have variations within these variables, we cannot advise either of the exercise intensities over the other; consequently, women may choose either LMI or HI exercise according to their own preferences.

## Data Availability Statement

The original contributions presented in the study are included in the article/Supplementary Material, further inquiries can be directed to the corresponding author/s.

## Ethics Statement

The studies involving human participants were reviewed and approved by Regional Ethical Review Board in Uppsala (Dnr 2014/249). The patients/participants provided their written informed consent to participate in this study.

## Author Contributions

AB: responsible for the writing process and statistical analyses throughout the whole process. LB, TR, and ID: planning, writing, discussing findings and part of the writing process, and editing the final manuscript. AS: advising regarding statistical matters and editing the final manuscript. KN: editing and feedbacks on the manuscript. SB: conceptualization, feedbacks and discussions throughout the whole writing process, and editing the final manuscript. All authors contributed to the article and approved the submitted version.

## Funding

This work was supported by the Swedish Cancer Society (Grant Nos. 150841 and 160483), the Swedish Research Council (Grant No. KDB/9514), the Nordic Cancer Union (2015), and the Oncology Department Foundations Research Fund in Uppsala (2016, 2017).

## Conflict of Interest

The authors declare that the research was conducted in the absence of any commercial or financial relationships that could be construed as a potential conflict of interest.

## Publisher's Note

All claims expressed in this article are solely those of the authors and do not necessarily represent those of their affiliated organizations, or those of the publisher, the editors and the reviewers. Any product that may be evaluated in this article, or claim that may be made by its manufacturer, is not guaranteed or endorsed by the publisher.

## Supplementary Material

Supplementary Figure S1 Adherence to the endurance training for participants < and > 61 years, and within the two exercise intensities. LMI, low-to-moderate intensity; HI, high intensity; * = *p* < 0.01. Means in % with 95% confidence interval.

Supplementary Table S1 General linear model regression including the eight models. All the seven models were adjusted for baseline VO_2_peak and hospital was included as a covariate.
