# Supplementary figures and images for: Exploring Moderators of the Effect of High vs. Low-to-Moderate Intensity Exercise on Cardiorespiratory Fitness During Breast Cancer Treatment – Analyses of a Subsample From the Phys-Can RCT
Source: Front Sports Act Living. 2022 Jul 12;4:902124. doi: 10.3389/fspor.2022.902124 (PMC9314879; doi:10.3389/fspor.2022.902124)

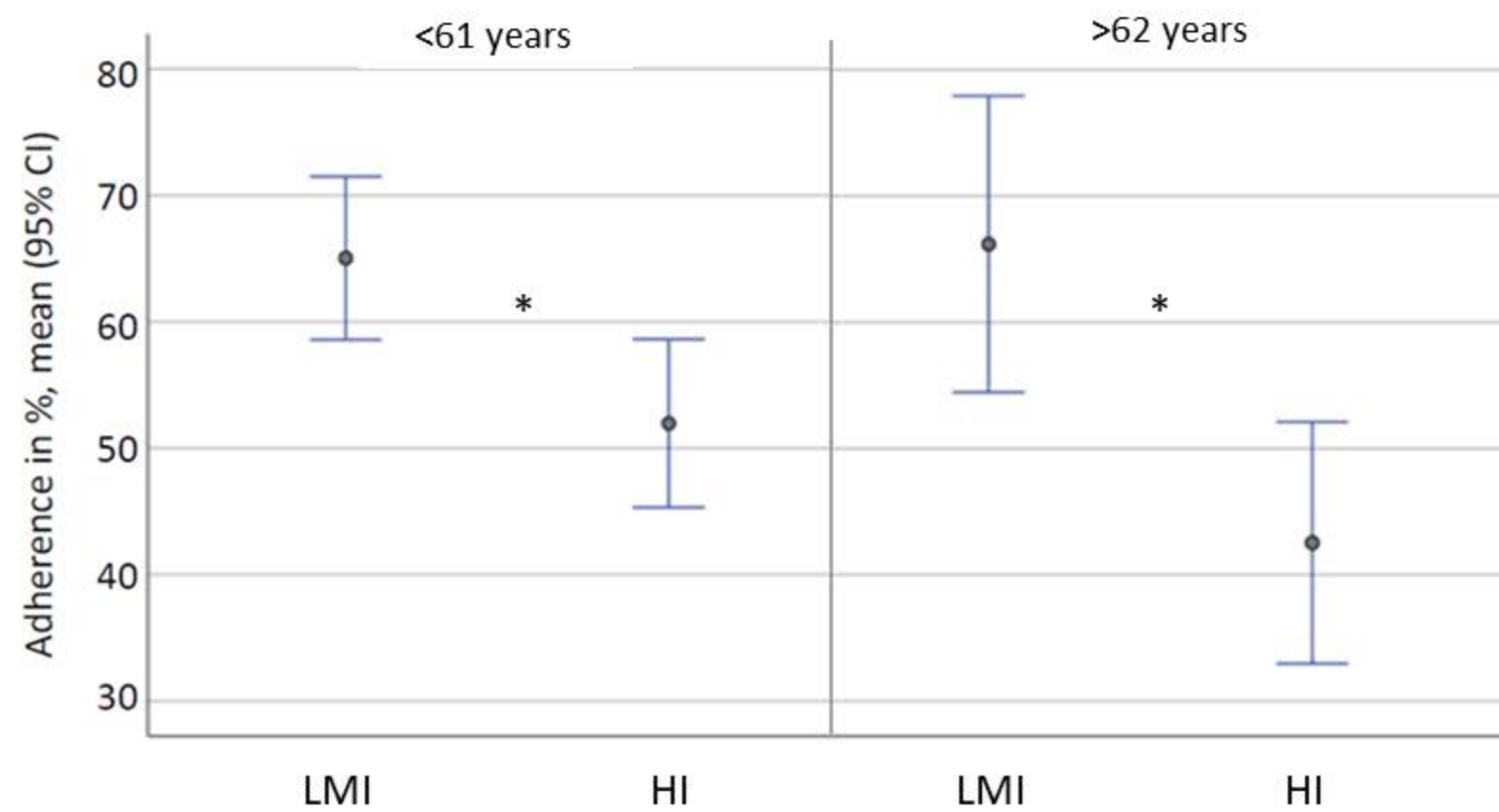

Supplement: Supplementary Figure S1 — Adherence to the endurance training for participants < and > 61 years, and within the two exercise intensities. LMI, low-to-moderate intensity; HI, high intensity; * = p < 0.01. Means in % with 95% confidence interval. [file Image_1.pdf]
