# Supplementary material for: Exploring Moderators of the Effect of High vs. Low-to-Moderate Intensity Exercise on Cardiorespiratory Fitness During Breast Cancer Treatment – Analyses of a Subsample From the Phys-Can RCT
Source: Front Sports Act Living. 2022 Jul 12;4:902124. doi: 10.3389/fspor.2022.902124 (PMC9314879; doi:10.3389/fspor.2022.902124)
Supplement: Supplementary Table S1 — General linear model regression including the eight models. All the seven models were adjusted for baseline VO2peak and hospital was included as a covariate. [file Table_1.pdf]

Suppl. table 1. General linear model regression including the eight models. All the seven models were adjusted for baseline VO_2peak and hospital was included as a covariate.

	Potential moderator variable	Coefficients, *b* (95% CI), p-value	Coefficients, $b_{interaction}$ (95% CI), p-value
Main model	*Constant*	4.92 (2.82, 7.01), <0.01	
	Intervention group LMI	-0.40 (-1.26, 0.46), 0.36	
	Intervention group HI (*ref*)	-	
	Linköping	0.66 (-0.52, 1.84), 0.27	
	Lund	-1.44 (-2.41, -0.46), <0.01	
	Uppsala (*ref*)	-	
	Baseline VO_2peak	0.84 (0.78, 0.90), <0.01	
1	Age, years	0.06 (0.01, 0.12), 0.04	
	Age*intervention		-0.08 (-0.16, 0.01), **0.06**
2	BMI, weight in kg/height in m^2	-0.10 (-0.24, 0.04), 0.14	
	BMI*intervention		-0.04 (-0.24, 0.15), 0.65
3	Physical fatigue, MFI	0.07 (0.78, 0.92), 0.35	
	Physical fatigue *intervention		-0.01 (-0.23, 0.21), 0.94
4	Chemotherapy treatment (no chemo.)	1.80 (0.54, 3.06), <0.01	
	Chemo. treatment*intervention (no chemo.*LMI)		0.03 (-1.77, 1.83), 0.97
5	MVPA, hours/wk	0.66 (-0.09, 1.41), 0.09	
	MVPA*intervention		-0.61 (-1.57, 0.34), 0.21
6	Baseline CRF (VO_2peak)	0.80 (0.71, 0.88), <0.01	
	Baseline CRF*intervention		0.09 (-0.03, 0.21), 0.15
7	Exercise adherence (<median)	-1.87 (-3.12, -0.62), <0.01	
	Exercise adherence*intervention (<median adh.*LMI)		1.63 (-0.12, 3.38), **0.07**

Abbreviations: BMI=Body Mass Index; MFI=Multidimensional Fatigue Inventory; MVPA=Moderate-to-vigorous physical activity; treatment=cytostatic vs no cytostatic; CRF=cardiorespiratory fitness; LMI=low-to-moderate intensity; adh.=adherence. Significance level was set to p<0.10 for further exploration.
